# Biomechanical and Parenchymal Determinants of Pain Perception During Mammography: Three-Dimensional Biometric Measurements and the Need for Personalized Compression

**DOI:** 10.3390/diagnostics16121819

**Published:** 2026-06-12

**Authors:** Abdulkadir Eren, Emrah Karatay, Irmak Durur Subasi

**Affiliations:** 1 Department of Radiology, Istanbul Medipol University Mega University Hospital, Istanbul 34214, Turkey; aeren@medipol.edu.tr; 2 Department of Radiology, Sultan 2. Abdulhamid Han Training and Research Hospital, Istanbul 34668, Turkey; 3 Department of Radiology, Institute of Senology, Acibadem Atakent University Hospital, Acibadem MAA University, Istanbul 34303, Turkey; irmak.subasi@acibadem.edu.tr

**Keywords:** mammography, pain, breast density, BI-RADS, biomechanics, personalized compression, biometric measurement

## Abstract

**Background/Objectives**: Standard mechanical compression applied during screening mammography is a primary barrier that reduces patient compliance. Current guidelines attempt to standardize compression based solely on the one-dimensional “breast thickness” measured by the device. This study aimed to investigate the effects of three-axis anatomical breast dimensions, applied compression force, menstrual cycle phases, and BI-RADS breast density patterns on pain scores during mammography within a comprehensive biomechanical model. **Methods**: This retrospective cohort study included 443 female patients who underwent routine screening or diagnostic mammography. Patients with a history of breast implants, lactation, or prior breast surgery that could alter tissue biomechanics were excluded. Maximum pain scores (1–10 on a Visual Analog Scale [VAS]) were recorded. Transverse, anteroposterior, and superoinferior breast biometric measurements for each patient were calculated using advanced radiological workstations. Data were analyzed using One-Way ANOVA and Multiple Linear Regression (OLS) models. **Results**: The mean age of the participants was 49.7 ± 9.4 years, the mean applied compression force was 62.4 ± 10.3 N, and the mean pain score was 2.03 ± 2.12 (range: 1–10). The multiple linear regression analysis was statistically significant overall (F = 2.516, *p* = 0.015). Having a BI-RADS Type D (extremely dense) breast pattern was identified as the strongest independent factor associated with an increased pain score (*p* = 0.082, coefficient = 1.219). Age showed a trend toward a negative effect on pain (*p* = 0.072), while compression force showed a trend toward a positive effect (*p* = 0.067). Conversely, breast thickness (*p* = 0.231) and the three-dimensional mean breast size index (*p* = 0.568) demonstrated no independent predictive power. The menstrual cycle phase did not reach independent significance in the multivariate regression model (*p* = 0.117); however, non-parametric univariate analysis revealed a significant difference in pain across hormonal groups (Kruskal–Wallis H = 10.04, *p* = 0.039), with actively menstruating and luteal-phase women reporting higher pain than menopausal women. **Conclusions**: The pain experienced during mammography depends on the internal fibroglandular architecture (elasticity and stiffness) of the tissue rather than its external volumetric dimensions. Notably, neither device-measured breast thickness nor manually calculated three-dimensional breast dimensions independently predicted pain, challenging the widespread assumption that breast size drives mammographic discomfort. “One-size-fits-all” or thickness-based compression strategies should be abandoned in routine practice. Instead, “personalized compression” protocols that prioritize patient comfort without compromising image quality should be developed, particularly for younger patients and those with BI-RADS Type D, and to a lesser extent Type C, density patterns.

## 1. Introduction

Breast cancer is the most commonly diagnosed malignancy among women globally and remains a leading cause of cancer-related mortality [1]. Full-field digital mammography (FFDM) and digital breast tomosynthesis (DBT) are currently considered the gold standards for detecting microcalcifications and early-stage lesions in asymptomatic populations [2]. The fundamental physical requirement for a successful mammographic examination is the mechanical compression of the target tissue between the X-ray tube and the detector. This compression reduces tissue superimposition to enhance lesion visibility, maximizes geometric sharpness, lowers radiation dose and secondary scatter, and prevents artifacts caused by patient motion or respiration [3,4].

However, this mechanical necessity comes with a significant clinical cost: pain. Current literature and survey-based epidemiological studies indicate that 40% to 75% of women undergoing mammography experience discomfort, with approximately 10% to 15% describing the pain as “severe and intolerable” [5,6]. The pain experience is not merely a momentary physical discomfort; it is one of the most substantial psychological barriers that reduces future screening compliance, delays screening, and ultimately undermines the goals of reducing breast cancer mortality [7].

Today, mammography units typically adjust compression based on the technician’s manual judgment, the device’s automated “thickness” reading, or predefined maximum force limits (e.g., between 100 and 200 N according to European reference guidelines) [8]. Nevertheless, these “one-size-fits-all” compression strategies ignore the heterogeneous biological structure and biomechanical properties of the breast. While many classical studies have investigated the relationship between compression force, one-dimensional breast thickness, and pain [9,10], there is a lack of multivariate models integrating pain with the three-dimensional volumetric anatomy of the breast (transverse, anteroposterior, and superoinferior axes), the patient’s current menstrual/hormonal status, and particularly the Breast Imaging Reporting and Data System (BI-RADS) parenchymal pattern, which reflects the internal fibroglandular architecture.

The major knowledge gap is that the causality attributed to the single-axis “breast thickness” parameter read by the device may be misleading. The mechanical resistance (stiffness) of the breast parenchyma changes radically based on the ratio of lipomatous to fibroglandular tissue [11]. Therefore, our hypothesis is that the true determinant of pain is not the geometric volume or thickness measured externally, but rather the intrinsic tissue elasticity reflected by the density pattern (the transition from Pattern A to Pattern D).

This study aimed to statistically model the independent effects of mammographic compression force, three-dimensional biometric measurements, age, and menstrual phase on pain perception in routine radiology practice—while excluding major confounding factors that could distort mechanical anatomy, such as lactation, surgical history, and silicone implants. Ultimately, this research seeks to provide a rational database for “personalized compression” applications in the literature.

## 2. Materials and Methods

### 2.1. Ethical Approval

This was a single-center, observational retrospective cohort study evaluating consecutive patients. This study protocol was approved by the Medical Faculty Ethics Committee of Istanbul Medipol University (E-10840098-202.3.02-5690, 5 May 2026).

### 2.2. Study Design, Patient Population and Exclusion Criteria

Data from 459 female patients who underwent routine screening or diagnostic FFDM in the radiology department between January 2024 and December 2025 were screened. Strict exclusion criteria were applied to eliminate conditions that could secondarily alter the natural biomechanical elasticity, volume, or nociceptive pain transmission of the breast: (1) patients with a history of breast augmentation (silicone/saline implants), (2) women who were actively lactating or had ceased breastfeeding within the past 6 months, (3) patients with a history of major breast surgery, such as mastectomy or lumpectomy, and (4) patients using chronic systemic analgesics, neuropathic medications (e.g., pregabalin or gabapentin), or antidepressants that could affect pain perception.

After excluding 16 patients with incomplete radiological or survey data, a total of 443 patients were included in the final statistical analysis.

### 2.3. Data Collection and Clinical Parameters

Patient demographic data (age) and clinical symptoms were obtained from the hospital information management system (HIMS). Menstrual cycle status was recorded from patient consent forms filled out prior to the procedure and categorized into five groups: 1 (Luteal phase/Premenstrual), 2 (Follicular phase/Postmenstrual), 3 (Perimenopause/Irregular bleeding), 4 (Postmenopause), and 5 (Active Menstruation).

Immediately following the procedure, each patient was asked to rate the maximum pain intensity experienced using a Visual Analog Scale (VAS)-like system ranging from 1 (only slight pressure sensation, no pain) to 10 (intolerable, severe pain). To prevent bias, these scores were recorded by an observer independent of the technician performing the mammogram.

### 2.4. Mammographic and Biometric Assessment

All mammographic examinations were performed using a high-end digital mammography system (Mammomat Revelation, Siemens Healthineers, Erlangen, Germany) equipped with wide-angle digital breast tomosynthesis (DBT) and automated compression pressure control features. Four projections were obtained: Craniocaudal (CC) and Mediolateral Oblique (MLO) for both breasts. For each projection, the compression force (in Newtons) and breast thickness (in millimeters) were automatically recorded from the DICOM metadata. The averages of the right/left and CC/MLO values were used for the analysis (Avg_Force, Avg_Thickness).

Three-Dimensional Biometric Measurements: Unlike traditional studies that rely solely on device-derived thickness, this study utilized advanced radiological PACS workstations (e.g., syngo.via, Siemens Healthineers) to manually measure the maximum anatomical dimensions of each breast across three axes: transverse, anteroposterior, and superoinferior. The average of these three values was calculated to create an “Average Size Index” for each patient.

Breast Density: The parenchymal composition was classified by an expert breast radiologist with at least 10 years of experience who was blinded to the patients’ pain scores, according to the 5th Edition of the ACR BI-RADS criteria: Pattern A (Almost entirely fatty), Pattern B (Scattered areas of fibroglandular density), Pattern C (Heterogeneously dense), and Pattern D (Extremely dense).

### 2.5. Statistical Analysis

Statistical analyses were performed using the SciPy (v1.7.3), Pandas (v1.3.5), and Statsmodels (v0.13.2) libraries in the Python (v3.9) ecosystem. The normality of variables was assessed using visual histograms, skewness/kurtosis, and Shapiro–Wilk tests. Continuous variables were expressed as mean ± standard deviation, while categorical variables were presented as frequencies and percentages (%). The main effects of categorical independent variables (BI-RADS Pattern, Menstrual Phase) on the pain scores were evaluated using One-Way Analysis of Variance (ANOVA). Additionally, the non-parametric Kruskal–Wallis H test was employed for categorical variables with imbalanced group distributions, such as the menstrual cycle phases. To determine the simultaneous and independent predictive effects of the independent variables (Age, Mean Force, Mean Thickness, Mean Size Index, and Breast Pattern) on the pain score, a Multiple Linear Regression (Ordinary Least Squares—OLS) model was constructed. Dummy variables were created for the categorical “Pattern” variable, with Pattern A serving as the reference. Statistical significance was set at α < 0.05, and *p*-values between 0.05 and 0.10 were reported as borderline trends.

## 3. Results

### 3.1. Demographic and Descriptive Findings

The mean age of the 443 patients meeting the study criteria was 49.71 ± 9.45 years (Range: 35–80). The mean compression force applied during the examinations was 62.39 ± 10.26 N, and the mean breast thickness recorded by the sensors was 56.57 ± 10.97 mm. The mean breast biometric size index measured via the PACS workstation was 17.25 ± 2.39 cm. The overall mean pain score (VAS) reported by the population was 2.03 ± 2.12, with a median value of 1. According to the radiological assessment, the majority of the population had high tissue density; Pattern D (Extremely Dense) had the highest frequency at 45.3% (*n* = 201), followed by Pattern C (36.7%), Pattern B (15.1%), and Pattern A (2.7%).

Regarding the hormonal/menopausal status of the study population, 182 patients (41.1%) were classified as postmenopausal (Group 4), representing a substantial proportion of the cohort. The remaining 261 patients (58.9%) were premenopausal or perimenopausal, categorized as: luteal phase (Group 1: *n* = 89, 20.1%), follicular phase (Group 2: *n* = 72, 16.3%), perimenopausal with irregular bleeding (Group 3: *n* = 58, 13.1%), and actively menstruating (Group 5: *n* = 42, 9.5%). The high proportion of postmenopausal women, who typically exhibit fatty involution and lower tissue stiffness, is consistent with the predominantly dense BI-RADS distribution observed and represents an important confounding variable to consider in the interpretation of density-pain associations. This distribution is detailed in Table 1.

### 3.2. Univariate Analysis of Categorical Factors

**Figure 1:**
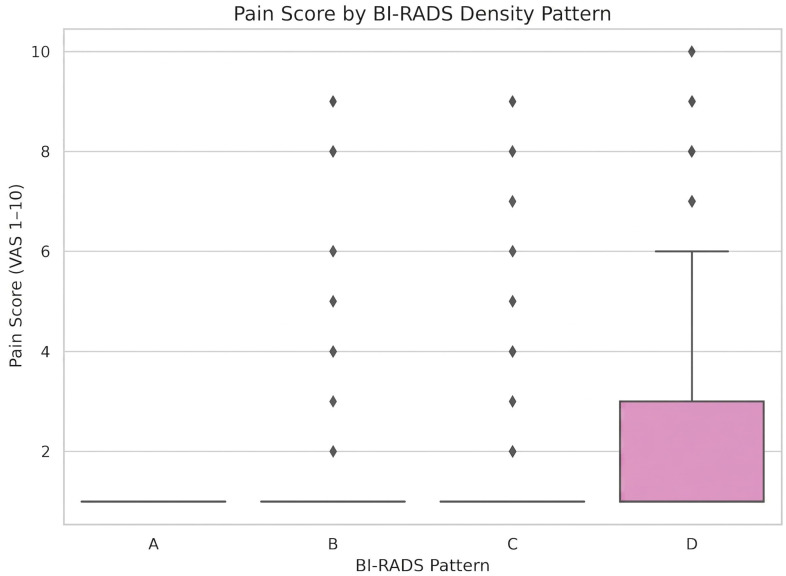
Box-and-whisker plot demonstrating the distribution of pain scores according to the ACR BI-RADS breast density patterns. A statistically significant variance was observed (ANOVA, *p* = 0.014), with patients having extremely dense breasts (Pattern D) reporting the highest median pain scores and upper quartiles.

A statistically significant variance was found in pain scores among the BI-RADS patterns representing the structural density of the breast (ANOVA F = 3.530, *p* = 0.014) (Figure 1). In subgroup analyses, a linear upward trend in mean pain scores was observed as tissue density shifted from Pattern A to Pattern D (Table 2).

**Figure 2:**
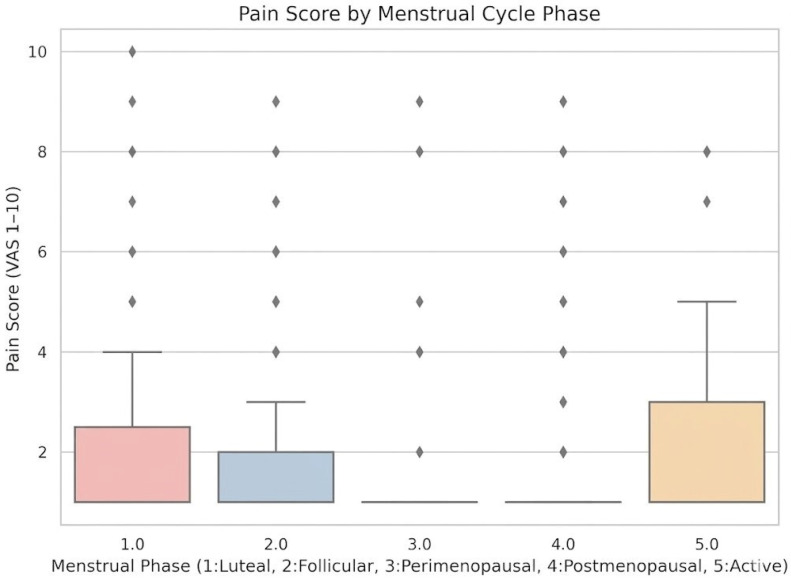
Box plot comparing pain scores across different phases of the menstrual cycle (1: Luteal, 2: Follicular, 3: Perimenopausal, 4: Postmenopausal, 5: Active Menstruation). Non-parametric analysis confirmed a statistically significant difference in pain perception based on the hormonal phase when accounting for group imbalances (Kruskal–Wallis H = 10.04, *p* = 0.039).

Additionally, a non-parametric Kruskal–Wallis test revealed a statistically significant difference in pain scores reported by patients across different phases of the menstrual/menopausal cycle (H = 10.04, *p* = 0.039) (Figure 2).

### 3.3. Multivariate Regression Analysis

**Figure 3:**
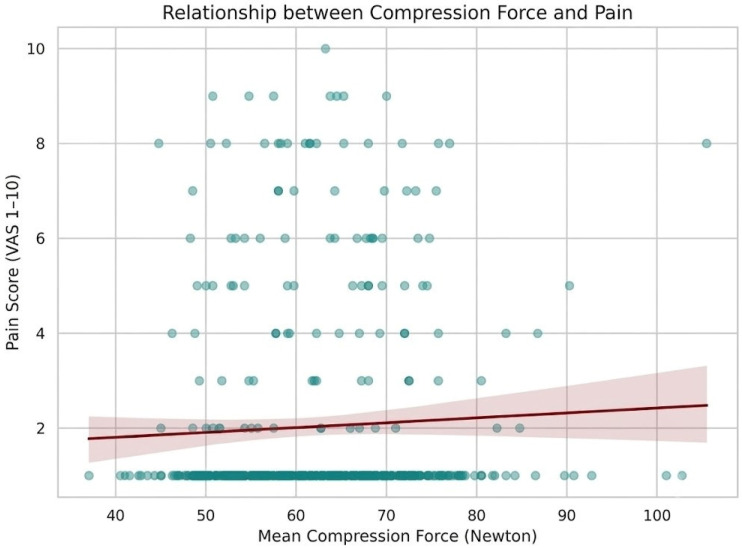
Scatter plot illustrating the relationship between applied mechanical compression force (Newtons) and pain scores. A borderline positive trend is visible (*p* = 0.067), suggesting that increased mechanical stress moderately correlates with higher pain perception. The solid red line indicates the linear regression trend line, and the shaded area represents the 95% confidence interval for the estimate.

**Figure 4:**
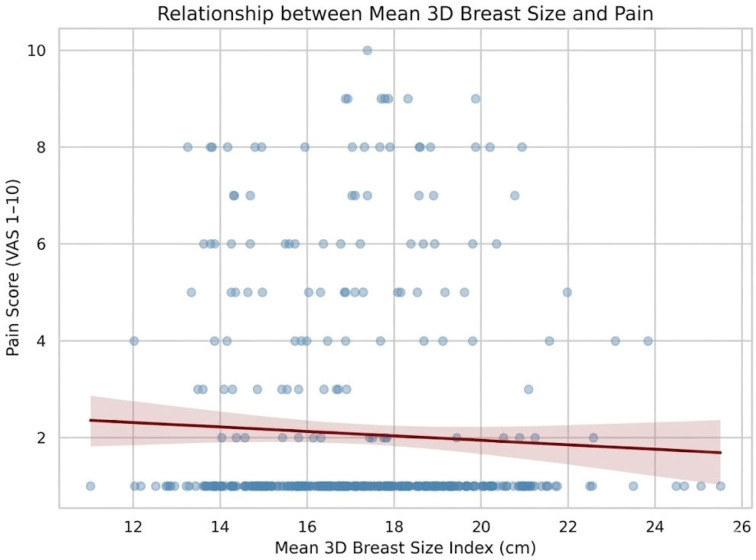
Scatter plot with a linear regression trendline showing the relationship between the 3D mean breast size index (cm) and reported pain scores. No significant correlation was found (*p* = 0.568), indicating that gross volumetric size does not independently predict pain.

**Figure 5:**
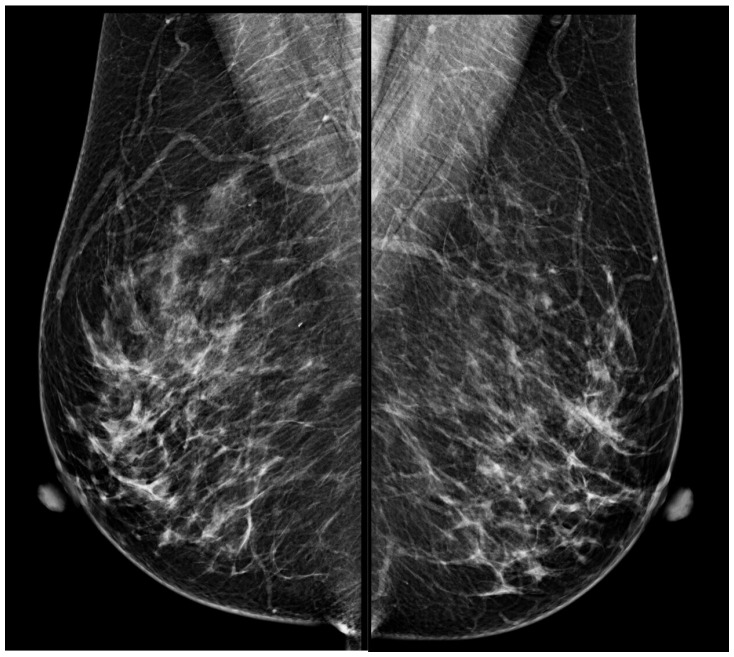
Synergistic Impact of Biomechanical and Hormonal Factors on Mammography Pain. Bilateral MLO mammograms of a 45-year-old patient with extremely dense breast parenchyma (BI-RADS Pattern D). Despite the application of varying mechanical compression forces (range: 56–74 N) and recorded breast thicknesses (range: 60–69 mm), the patient reported a maximum pain score (VAS 10/10). This clinical presentation highlights the “Biomechanical Paradox”, where intrinsic tissue stiffness in dense breasts, exacerbated by the hormonal congestion and edema typical of the luteal phase, significantly increases nociceptive sensitivity. The case underscores that pain perception is not merely a function of applied force, but a complex interaction between tissue elasticity and the patient’s hormonal cycle, supporting the necessity for personalized compression protocols.

**Figure 6:**
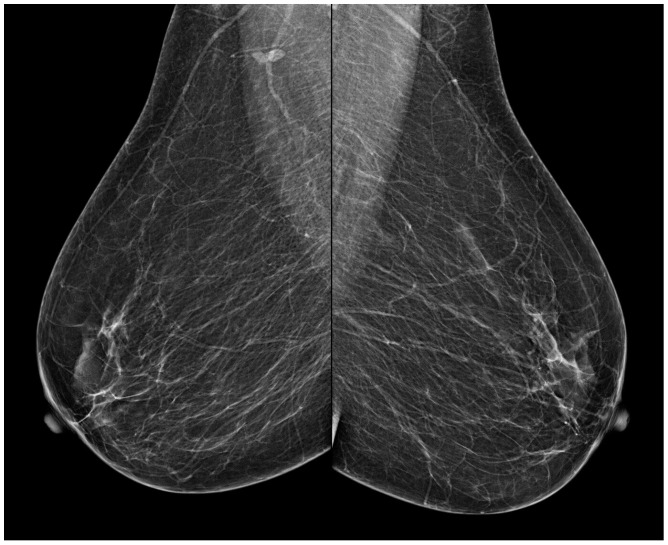
Bilateral MLO mammograms of a 61-year-old postmenopausal patient with a BI-RADS Pattern A (predominantly fatty) breast structure. Despite the application of high compression forces (reaching up to 70 N) and breast thicknesses ranging between 37 and 47 mm, the patient reported a minimal pain score (VAS 1/10). This case demonstrates that postmenopausal fatty involution significantly reduces tissue resistance and nociceptive sensitivity. In contrast to younger patients with dense breasts, high mechanical force does not necessarily translate to high pain perception in fatty tissue, further validating the need for density-based personalized compression rather than a one-size-fits-all force standard, emphasizing that breast density is a more reliable predictor of procedural pain than the magnitude of mechanical force.

To predict the pain score (1–10) as the dependent variable, an OLS regression model incorporating patient age, applied force, breast thickness, 3D breast size index, and breast pattern (using dummy variables) was constructed. The overall model was statistically significant (Model F = 2.516, *p* = 0.015). The independent coefficients and significance levels of the variables in the model are summarized below (Table 3):

1. Breast Pattern: Compared to the reference Pattern A, having an extremely dense Pattern D was identified as the factor that most robustly and independently increased the pain score in the regression model (*p* = 0.082, Coefficient = +1.219; borderline significance but highest effect size).
2. Age Factor: There was a negative relationship between patient age and perceived pain. As age increased, the reported pain score tended to decrease (*p* = 0.072, Coefficient = −0.024).
3. Applied Force (N): An increase in the mechanical compression force applied to the breast tissue showed a trend toward positively influencing the pain score (*p* = 0.067, Coefficient = +0.020). (Figure 3).
4. Biometric Volume and Thickness: In the regression model, neither the single-axis “Breast Thickness (mm)” measured by the device (*p* = 0.231, Coefficient = −0.014) nor the multi-axis “Mean Breast Size” calculated manually from radiological workstations (*p* = 0.568, Coefficient = +0.038) possessed independent predictive power over pain. (Figure 4). In other words, merely having a “large” or “thick” breast did not explain the pain when tissue density and age were held constant (Figure 5 and Figure 6).

## 4. Discussion

Pain associated with mammography is the Achilles’ heel of breast cancer screening. In this study, utilizing a cohort free from factors that alter biomechanical architecture, such as breast implants or lactation, we demonstrated that the deterministic foundation of pain during mammography is not merely the mechanical compression force applied by the device, but rather the “Breast Density Pattern (BI-RADS)” which dictates the intrinsic elasticity of the tissue. Our findings highlight the inadequacy of standardized, thickness-based compression protocols in radiology practice and offer a new perspective centered on personalized tissue biomechanics.

The most striking finding of our study is that having a BI-RADS Type D parenchymal pattern emerged as the strongest independent factor augmenting pain. This is directly related to the physics and neurophysiology underlying mammographic compression. Breast parenchyma is a heterogeneous amalgamation of adipose and fibroglandular tissue. MR Elastography studies and ex vivo biomechanical tests reveal that the Young’s modulus of fibroglandular tissue is dramatically higher than that of adipose tissue [12,13]. Stated simply, dense tissue exhibits significantly greater resistance to mechanical stress (stiffness).

When a technician approaches a Pattern A (fatty, soft) and a Pattern D (dense, stiff) breast with similar pressure to achieve the same image clarity, the Cooper’s ligaments and stromal structures in the Pattern D tissue are subjected to far greater mechanical tension. This mechanical stress easily surpasses the threshold of nociceptive A-delta and C nerve fibers within the tissue, resulting in higher pain scores [14]. De Groot et al. [15] argued that pressure-based compression reduces pain compared to standard force application. Our data advance this thesis further, demonstrating that the “type of tissue” targeted by the force explains the variance in pain even more than the force itself.

Furthermore, our data reveals a striking biomechanical paradox that reinforces the profound mediating role of breast density. According to our measurements, patients with an extremely dense Pattern D parenchyma received significantly less absolute compression force (mean 60.0 N) and possessed smaller transverse breast dimensions (mean 17.5 cm) compared to patients with a fatty Pattern A density, who received the highest compression force (mean 70.5 N) on larger breasts (mean 22.0 cm). Despite being subjected to the lowest mechanical force and having the smallest physical volume, women with Pattern D reported the highest pain scores. This paradox definitively uncouples mastalgia from the raw mechanical output of the mammography unit. It proves that the true nociceptive stimulus is not the absolute Newton force applied, but rather the failure of dense, inelastic fibroglandular tissue to accommodate even minimal mechanical stress. The rigidity of the parenchymal substrate converts minimal external compression into severe internal nociceptive signaling.

Many classical papers in the literature assert that as breast thickness increases, pain also increases [16]. However, the major flaw in these studies is their failure to adjust for breast thickness and breast density within the same regression model. In our study, utilizing a methodology rarely seen in the literature, not only was the device’s automated “thickness” data used, but a 3-axis anatomical size index was calculated via advanced workstations and included in the model. When breast density was controlled for, neither breast thickness (*p* = 0.231) nor the volumetric size index (*p* = 0.568) had any significant independent effect on pain. This indicates that the “thickness” effect observed in previous studies may actually be a confounding bias reflecting density. That is, a large breast does not inherently mean it will hurt more; the determinant of pain is not the gross volume, but how densely it is packed with glandular stroma.

Our study found a tendency for pain scores to decrease with advancing age (Coefficient: −0.024, *p* = 0.072). This aligns perfectly with the biological process of menopausal involution that occurs with aging [17]. As a woman ages, stiff and dense fibroglandular tissue is progressively replaced by mechanically compliant and soft adipose tissue. The increased pain perception observed in younger populations can be explained not only by cellular density but potentially by a higher general pain perception threshold and elevated anxiety levels in younger women [18].

Interestingly, while the overall multiple regression model demonstrated significant predictive validity (*p* = 0.015), individual predictors such as age, force, and Pattern D achieved only borderline significance. This reflects the inherent multicollinearity between breast density, age, and mechanical compression; when these highly intertwined biological and mechanical predictors enter the model simultaneously, shared variance causes individual coefficients to lose strict significance. Rather than a statistical weakness, this shared variance strongly reinforces our premise: density, age, and force are not isolated variables but function as a highly correlated biomechanical network.

Furthermore, re-evaluating the hormonal spectrum using non-parametric analysis to account for group imbalances revealed a statistically significant difference in pain across menstrual phases (Kruskal–Wallis H = 10.04, *p* = 0.039). Women in the luteal phase and active menstruation reported higher pain scores compared to postmenopausal women, which is consistent with the high prevalence of cyclical mastalgia during these hormonally active periods [19]. The hormonal influence on pain operates primarily through its long-term effect on parenchymal composition (density classification) rather than solely through short-term cycle-related fluid shifts [20]. This confirms that cyclical hormonal nociception within the dense fibroglandular matrix is a key driver of mastalgia.

Current European and American reference guidelines establish strict force limits for compression [8]. However, the results of our study suggest that the “one-size-fits-all” approach is obsolete. In future radiology practice, mammography systems should be capable of instantly determining the patient’s BI-RADS density utilizing Artificial Intelligence—either from a previous screening file or a low-dose pre-shot—and automatically optimizing the stopping thresholds of compression motors according to this tissue stiffness (different for Pattern A vs. Pattern D) (Personalized Compression) [21]. However, a recognized practical constraint of this density-informed approach is that for first-time screening patients, a priori BI-RADS density classification is not readily available. Unless estimated via a low-dose pre-shot, the absence of prior radiological records poses a logistical challenge in seamlessly implementing personalized compression protocols for initial baseline screenings. The evolution of technology in this direction is the most critical intervention to prevent women, especially those who are young and possess Type C–D density, from avoiding screening programs.

Furthermore, the recent literature evaluating different compression parameters and flexible paddle designs also emphasizes the critical impact of these procedural variables on mitigating patient discomfort [22,23,24]. Beyond technological solutions, an immediately practicable approach deserves emphasis: manually applied or slowly incremented compression, in which the patient retains control over the maximum compression tolerated, represents a low-cost intervention with considerable potential benefit for all apprehensive women, regardless of density pattern. The fear associated with automatic rapid compression—over which the patient has no agency—is a distinct and likely underappreciated contributor to the pain scores recorded in studies such as this one. Encouraging patient-controlled compression not only addresses the psychological component of pain but also aligns with a patient-centered care philosophy. Our data showing that force was associated with pain (*p* = 0.067) further support the rationale for slow, controlled compression as a broadly applicable strategy rather than one reserved exclusively for dense-breast patients.

Our study has certain limitations. Although the analyses were conducted on a large patient series, the study was performed at a single center. Pain is a subjective experience, and psychological anxiety factors—such as patients’ educational background or history of prior breast biopsies—were not evaluated. Secondly, variations in manual compression habits among different technicians were not included in the model; however, obtaining data from technicians who underwent standardized training at a single center minimized this variance. Thirdly, the use of exogenous hormones (e.g., oral contraceptives or hormone replacement therapy) was not recorded, which may act as an unmeasured confounder. Fourthly, breast density was assessed by a single expert radiologist; inter-reader variability was not formally evaluated, which may limit the reproducibility of density classification. Finally, our pain assessment relied on a single NRS score immediately after the procedure, without formally distinguishing between pre-existing cyclical mastalgia and strictly procedure-related compression pain.

Another limitation of our study is the highly skewed distribution of breast density patterns within the cohort. A large majority of the patients exhibited Pattern D (45.3%), whereas only a small fraction exhibited Pattern A (2.7%). This quantitative imbalance reduces the statistical power of comparative regression analyses between these specific extremes. Future studies should aim for a deliberately balanced prospective recruitment across all BI-RADS categories to enhance the robustness of these statistical inferences.

An important conceptual limitation warrants explicit acknowledgment: the ACR BI-RADS “density” classification, as employed in this study, quantifies radiographic opacity—the approximate ratio of fibroglandular tissue to fat as visualized on mammography—rather than true tissue density in terms of mass per unit volume. It does not differentiate between the intralobular and extralobular collagen fractions, nor does it precisely measure the ratio of glandular tissue to the stromal collagen component. Furthermore, there is documented intra-reader and inter-reader variability in BI-RADS classifications, which may be compounded by differences in image contrast algorithms across mammography manufacturers. Single-reader BI-RADS assessment, while presumably internally consistent within our dataset, may contain personal bias and limits generalizability. Future studies incorporating automated AI-based density quantification tools or volumetric density measurements would provide more objective and reproducible assessments. Additionally, although this study did not formally assess psychological anxiety, the manner of compression application—specifically, the use of automatic versus manually controlled compression—may itself be a source of pain-related fear and must be acknowledged as an unmeasured factor influencing pain perception scores.

## 5. Conclusions

The primary determinant of the pain experienced during mammography is not the external dimensions of the breast or the thickness measured by the device; it is the mechanical resistance tied to the internal fibroglandular architecture, namely the BI-RADS density pattern. Patients with dense breast parenchyma (Types C and D) and those who are relatively young represent the group at highest risk for severe pain due to mechanical stress. Radiology technicians should focus not only on device sensors or physical thickness but also on the patient’s tissue pattern when applying compression. It is imperative that clinical guidelines be updated toward “personalized compression” goals that prioritize tissue elasticity and biomechanics.

Critically, compression force was associated with higher reported pain scores (*p* = 0.067), while breast size and thickness showed no independent predictive value, challenging the widespread assumption that breast volume drives mammographic discomfort. Pain is a highly subjective experience modulated by psychological factors, including anxiety, fear of automated compression, and prior painful experiences; future studies should incorporate formal psychological assessments. The benefits of personalized compression should ideally extend to all women rather than being restricted to those with high-density patterns. For apprehensive patients, manually controlled or gradually applied compression that allows the patient to exercise control over the process may substantially reduce fear-related pain, independent of breast density classification. The BI-RADS density pattern, while an imperfect proxy for true tissue biomechanics due to its reliance on radiographic opacity rather than direct measurement of tissue stiffness or glandular composition, remains a clinically accessible and practically useful variable for guiding compression protocols until more objective, real-time tissue characterization tools become widely available.

## Figures and Tables

**Table 1:** Patient Demographics, Mammographic, and Biometric Characteristics.

Variables	Values (*N* = 443)
Age (years), Mean ± SD (Range)	49.71 ± 9.45 (35–80)
Mean Compression Force (Newton), Mean ± SD	62.39 ± 10.26
Mean Breast Thickness (mm), Mean ± SD	56.57 ± 10.97
Mean 3D Breast Size Index (cm), Mean ± SD	17.25 ± 2.39
Maximum Pain Score (VAS 1–10), Mean ± SD (Median)	2.03 ± 2.12 (1.0)
BI-RADS Density Pattern, *n* (%)	
- Pattern A (Almost entirely fatty)	12 (2.7%)
- Pattern B (Scattered fibroglandular density)	67 (15.1%)
- Pattern C (Heterogeneously dense)	163 (36.8%)
- Pattern D (Extremely dense)	201 (45.4%)

**Table 2:** Comparison of Pain Scores by Categorical Variables.

Categorical Variables	Test Statistic	*p*-Value	Significance
BI-RADS Breast Density Pattern (A, B, C, D)	F = 3.530	0.014 *	Significant
Menstrual Cycle Phase (5 Phases)	H = 10.040	0.039 *	Significant

* *p* < 0.05 indicates statistical significance. (F: ANOVA test statistic, H: Kruskal–Wallis test statistic).

**Table 3:** Multiple Linear Regression Model Predicting Mammographic Pain Score.

Independent Variables	Coefficient (β)	Standard Error	t-Value	*p*-Value	95% CI
Age	−0.024	0.013	−1.806	0.072 ^	[−0.051, 0.002]
Applied Compression Force (N)	0.021	0.011	1.834	0.067 ^	[−0.001, 0.043]
Breast Thickness (mm)	−0.014	0.012	−1.199	0.231	[−0.038, 0.009]
3D Mean Breast Size Index	0.038	0.067	0.571	0.568	[−0.093, 0.169]
BI-RADS Pattern B (Ref: Pattern A)	0.979	0.675	1.451	0.148	[−0.348, 2.306]
BI-RADS Pattern C (Ref: Pattern A)	0.693	0.666	1.041	0.299	[−0.616, 2.003]
BI-RADS Pattern D (Ref: Pattern A)	1.219	0.699	1.744	0.082 ^	[−0.155, 2.594]

Model Summary: F = 2.516, *p* = 0.015. ^ *p* < 0.10 indicates a borderline statistical trend.

## Data Availability

The datasets generated and/or analyzed during the current study are not publicly available due to patient privacy and confidentiality agreements but are available from the corresponding author upon reasonable request.

## Abbreviations

The following abbreviations are used in this manuscript:

ACR	American College of Radiology
ANOVA	Analysis of Variance
BI-RADS	Breast Imaging Reporting and Data System
BPE	Background Parenchymal Enhancement
CC	Craniocaudal
DBT	Digital Breast Tomosynthesis
DICOM	Digital Imaging and Communications in Medicine
FFDM	Full-Field Digital Mammography
HIMS	Hospital Information Management System
IRB	Institutional Review Board
MLO	Mediolateral Oblique
N	Newton (Unit of Force)
OLS	Ordinary Least Squares
PACS	Picture Archiving and Communication System
SD	Standard Deviation
VAS	Visual Analog Scale
